# Correlation between c-Met and ALDH1 contributes to the survival and tumor-sphere formation of ALDH1 positive breast cancer stem cells and predicts poor clinical outcome in breast cancer

**DOI:** 10.18632/genesandcancer.148

**Published:** 2017-07

**Authors:** Yuka Nozaki, Shoma Tamori, Masahiro Inada, Reika Katayama, Hiromi Nakane, Osamu Minamishima, Yuka Onodera, Makoto Abe, Shota Shiina, Kei Tamura, Daichi Kodama, Keiko Sato, Yasushi Hara, Ryo Abe, Ryoko Takasawa, Atsushi Yoshimori, Nariyoshi Shinomiya, Sei-ichi Tanuma, Kazunori Akimoto

**Affiliations:** ^1^ Department of Medicinal and Life Science, Faculty of Pharmaceutical Sciences, Tokyo University of Science, Chiba, Japan; ^2^ Department of Pharmacy, Faculty of Pharmaceutical Sciences, Tokyo University of Science, Chiba, Japan; ^3^ Department of Information Sciences, Faculty of Science and Technology, Tokyo University of Science, Chiba, Japan; ^4^ Research Institute for Biochemical Sciences, Tokyo University of Science, Chiba, Japan; ^5^ Translational Research Center, Research Institute for Science& Technology, Tokyo University of Science, Chiba, Japan; ^6^ Institute for Theoretical Medicine, Inc., Yokohama, Japan; ^7^ Department of Integrative Physiology and Bio-Nano Medicine, National Defense Medical College, Saitama, Japan

**Keywords:** ALDH1, breast cancer, CSC, c-Met

## Abstract

c-Met is a receptor-type tyrosine kinase, which is involved in a wide range of cellular responses such as proliferation, motility, migration and invasion. It has been reported to be overexpressed in various cancers. However, the role of c-Met in breast cancer stem cells (CSCs) still remains unclear. We herein, show that *c-Met* expression is significantly elevated in Basal-like type of breast cancer in comparison with other subtypes. High expression of *c-Met* strongly correlated with the expression of two CSC markers, *ALDH1A3* and *CD133* in breast cancers. In addition, breast cancers at tumor stage III-IV expressing both *c-Met*^high^ and *ALDH1A3*^high^ had poor prognosis. Furthermore, treatment with c-Met inhibitors (Crizotinib, Foretinib, PHA-665752 and Tivantinib) in MDA-MB157 cells with high c-Met protein expression resulted in significant suppression in cell viability, contrary to MDA-MB468 cells with low c-Met protein expression. These c-Met inhibitors also suppressed cell viability and tumor-sphere formation of ALDH1^high^ breast cancer cells with high c-Met expression. These results suggest that c-Met in ALDH1 positive CSCs seems to play an important role in breast cancer repopulation. Therefore, we conclude that c-Met is a potential therapeutic target in ALDH1 positive breast CSCs.

## INTRODUCTION

Breast cancer is one of the most common cancers occurring in women worldwide with 1.7 million new cases (25.2% of all cancers in women) and 0.5 million-cancer deaths (14.7% of all cancer death in women) according to an estimate from the International Agency for Research on Cancer (IARC) [1]. Breast cancer has been widely classified based on specific gene expression signature and receptor status. Based on PAM50 gene expression signature, breast cancer is categorized into six ‘'intrinsic’’ subtypes namely, Luminal A, Luminal B, HER2-enriched, Claudin-low, Basal-like, and Normal-like [2, 3, 4], of which, Basal-like type has poor prognosis [5]. Based on receptor status, breast cancer is categorized into estrogen receptor (ER)-positive type, progesterone receptor (PgR)-positive type, HER2 positive type, and triple-negative type (ER-negative, PgR- negative, HER2-negative) (TNBC). Among them, TNBC has the poorest prognosis. Notably, among 70-80% of Basal-like type of breast cancer has been reported to fall into TNBC category [6].

Tumors are comprised of population of cancer cells and distinct cancer stem cells (CSCs), which are largely undifferentiated tumorigenic cells with stem-like properties such as self-renewal and multipotency [7, 8]. Most CSCs are resistant to conventional anti-tumor treatments, chemo- and radio-therapies, which consequently leads tumor recurrence and metastasis. Therefore, the development of targeted therapies against CSCs is highly required to improve poor clinical outcome.

CSCs in breast tumor patients can be identified based on the expression of aldehyde dehydrogenase (ALDH) isoforms. ALDH1 has been reported to be enriched in CSCs of several cancer types, including breast cancer and is a potential CSC marker [9, 10, 11]. Among ALDH1 gene family, isoforms *ALDH1A1* and *ALDH1A3* are also known as CSCs markers in several cancers [11, 12, 13, 14]. Particularly, isoform *ALDH1A3* has been reported to contribute significantly to ALDH1 activity in breast cancer cells and its expression significantly correlates with cancer type, tumor grade and metastasis in breast tumor patients [15]. On the other hand, there are controversial results regarding the involvement of ALDH1 in breast cancer subtypes [16, 17, 18].

c-Met is a receptor-type tyrosine kinase, which is involved in wide range of cellular responses such as proliferation, motility, migration, invasion and tumor angiogenesis [19, 20]. c-Met has been reported to be highly expressed and aberrantly activated in variety of cancers [21, 22, 23]. High expression of c-Met correlating with the expression of CSCs markers such as CD133, CD44, and ALDH1 has also been reported [24, 25, 26]. Furthermore, c-Met protein has been reported to be involved in biological processes of head and neck, and pancreatic CSCs [26, 27]. However, the relationship of c-Met with ALDH1 positive CSCs in breast cancer subtypes still remains unclear.

In this study, we show that high expression of c-Met correlates with the expression of *ALDH1A3* in breast cancer. Patients with co-expression of *c-Met* and *ALDH1A3* at tumor stage III-IV showed poor clinical outcome. Furthermore, c-Met inhibitors suppressed the viability and tumor-sphere formation of ALDH1^high^ cells. These results suggest that c-Met is essential for the viability and tumor formation of ALDH1 positive breast CSCs. Therefore, c-Met protein is a promising therapeutic target for ALDH1 positive breast CSCs.

## RESULTS

### Correlation of *c-Met* with CSC markers at gene expression level in human breast cancers

**Figure 1:**
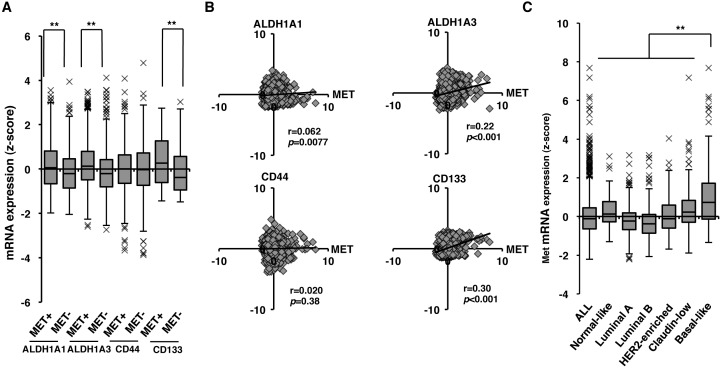
*c-Met* expression correlates with gene expression of human breast CSC markers **A.** Gene expression levels of *ALDH1A1*, *ALDH1A3*, *CD44*, and *CD133* with high (c-Met^+^) and low (c-Met^-^) *c-Met* expression in primary breast tumors. Values are shown as box-and-whisker plot (Tukey's test, **p < 0.01). **B**. Correlation of *c-Met* with *ALDH1A1*, *ALDH1A3*, *CD44*, and *CD133* in primary breast tumors. Values are shown as scattered plots. The coefficient of correlation (r) and the *p* value (*p*) are indicated. **C**. *c-Met* expression levels in breast cancer subtypes. Values are shown as box-and-whisker plot (Tukey's test, **p < 0.01).

To investigate the association of *c-Met* with CSC markers such as *ALDH1A1*, *ALDH1A3*, *CD44*, and *CD133* at gene expression levels in human breast cancers, we analyzed mRNA data and the clinical information of 1904 patients of breast cancers from cBioPortal for Cancer Genomics [28, 29]. As shown in Figure 1A, high expression of *c-Met* (MET^+^) correlated with expression of CSC markers, *ALDH1A1* (*p* < 0.001), *ALDH1A3* (*p* < 0.001), and *CD133* (*p* < 0.001) in breast cancers. In addition, scatter plots analysis also indicated that *c-Met* expression correlated with *ALDH1A1* (*p* = 0.0077), *ALDH1A3* (*p* < 0.001) and *CD133* expression (*p* < 0.001) (Figure 1B and Table 1). *c-Met* expression was also found to be associated with several undifferentiated markers, such as *KLF4*, *c-Myc*, *Notch1*, *Notch3*, and *BMI1* (Table 1). Next we examined the mRNA expression level of *c-Met* in the specific breast cancer subtypes. As shown in Figure 1C, *c-Met* mRNA was found to be enriched in Basal-like type in comparison with other subtypes, such as Normal-like, Luminal A, Luminal B, HER2-enriched, and Claudin-low.

**Table 1:** Correlation analysis between *c-Met* with cancer stem cell or undifferentiated markers in all stage, stage 0, I, II and stage III, IV of breast tumors

mRNA co-expression MET vs.	ALL Stage		Stage I-II		Stage III-IV	
	Pearson's Correlation	*p*-value	Pearson's Correlation	*p*-value	Pearson's Correlation	*p*-value
*ALDH1A1*	0.06	0.008	0.05	0.058	-0.03	0.736
*ALDH1A3*	0.22	<0.001	0.21	<0.001	0.40	<0.001
*CD44*	0.02	0.381	0.01	0.857	0.04	0.637
*CD133*	0.30	<0.001	0.31	<0.001	0.40	<0.001
*KLF4*	0.10	<0.001	0.11	<0.001	0.12	0.181
*MYC*	0.14	<0.001	0.15	<0.001	0.17	0.055
*NANOG*	-0.04	0.064	-0.02	0.560	0.01	0.895
*NOTCH1*	0.17	<0.001	0.16	<0.001	0.43	<0.001
*NOTCH3*	0.06	0.010	0.02	0.449	0.22	0.017
*OCT4*	0.02	0.325	0.00	0.888	0.35	<0.001
*SOX2*	0.00	0.882	0.01	0.730	-0.05	0.609
*STAT3*	-0.03	0.156	-0.05	0.078	-0.05	0.558
*BMI1*	-0.16	<0.001	-0.17	<0.001	-0.20	0.023

### Correlation of *c-Met* with *ALDH1A3* and *CD133* at gene expression level in breast cancer at tumor stage III-IV

**Figure 2:**
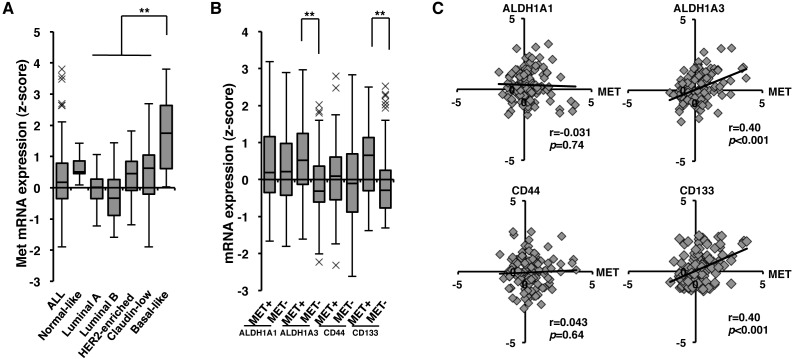
Correlation of *c-Met* with *ALDH1A3* and *CD133* at gene expression level in tumor stage III and IV of breast cancer **A.** *c-Met* expression levels in breast cancer subtypes of tumor stage III-IV. Values are shown as box-and-whisker plot (Tukey's test, **p < 0.01). **B**. Gene expression levels of *ALDH1A1*, *ALDH1A3*, *CD44*, and *CD133* with high (c-Met+) and low (c-Met -) *c-Met* expression at tumor stage III-IV. Values are shown as box-and-whisker plot (Tukey's test, **p < 0.01). **C**. Correlation of *c-Met* with *ALDH1A1*, *ALDH1A3*, *CD44*, and *CD133* at tumor stage III-IV. Values are shown as scattered plots. The coefficient of correlation (r) and the *p* value (*p*) are indicated.

Since overexpression of c-Met contributes to cancerous progression [21,22,23], we next examined *c-Met* expression at various tumor stages. Among early tumor stage lesions (0, I, II; *n* = 1279), 45% were c-Met^+^ (n = 573), contrary to 59% of c-Met^+^ (*n* = 74) at tumor late stage lesions (III, IV; *n* = 124). As c-Met^+^ tumor lesions were higher in tumor stage III-IV, in contrast with stage 0, I, and II, we next focused to analyze the relationship between *c-Met* gene expression and CSC markers in breast cancer subtypes at tumor stage III-IV. *c-Met* mRNA was found to be enriched in Basal-like type in comparison with other subtypes at stage III and IV (Figure 2A). As shown in Figure 2B, c-Met^+^ strongly correlated with ALDH1A3**^+^** (*p* < 0.001). c-Met^+^ also weakly associated with CD133^+^ (*p* = 0.0025). Scatter plots analysis also indicated that *c-Met* expression correlated with *ALDH1A3* (*p* < 0.001) and *CD133* expression (*p* < 0.001) (Figure 2C and Table 1). *c-Met* expression was also found to be associated with several undifferentiated markers, such as *Notch1*, *Oct4*, and *BMI1* (Table 1). These results indicate that c-Met plays important roles in ALDH1 and/or CD133 positive CSCs.

### Co-expression of *c-Met*^high^ and *ALDH1A3*^high^ indicated poor prognosis

**Figure 3:**
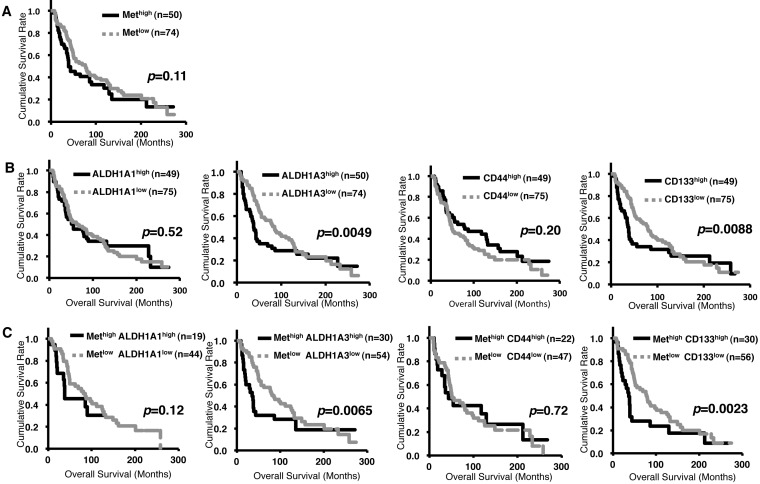
Co-expression of *c-Met* with *ALDH1A3* or *CD133* contributes to poor prognosis in breast cancer patients at tumor stage III-IV Kaplan-Meier Survival curves of human breast cancer at tumor stage III-IV. **A**. *c-Met* expression. **B**. *ALDH1A1, ALDH1A3, CD44* or *CD133* expression. **C**. *c-Met* and *ALDH1A1*, *ALDH1A3*, *CD44* or *CD133* expression.

Further, we next performed Kaplan-Meier analysis of *c-Met* and CSC markers at tumor stage III-IV. *c-Met*^high^ patients did not show poor prognosis (*p* = 0.11) (Figure 3A), whereas *ALDH1A3*^high^(*p* = 0.0049) and *CD133*^high^(*p* = 0.0088) showed poor prognosis (Figure 3B). Interestingly, co-expression of both *c-Met*^high^ with *ALDH1A3*^high^ (*p* = 0.0065), and with *CD133*^high^ (*p* = 0.0023) indicated poor prognosis (Figure 3C). These results indicate that c-Met plays important roles in cancerous progression and contributed to the poor prognosis in ALDH1 positive and/or CD133 positive breast CSCs. Since the role of c-Met in biological properties of CD133 positive CSCs is reported [24, 32, 33], hence, we focused on investigating the roles of c-Met in ALDH1 positive breast CSCs.

### c-Met inhibitors suppressed viability of ALDH1 positive CSCs

**Figure 4:**
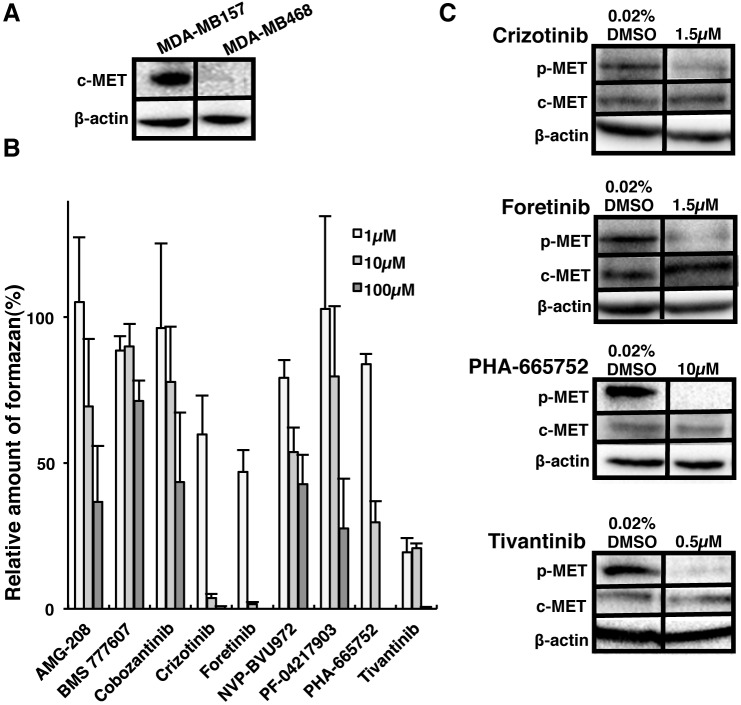
c-Met inhibitors suppressed cell viability and c-Met activation in Basal-like type of breast cancer cell lines **A.** c-Met expression in Basal-like type of breast cancer cell lines, MDA-MB157 and MDA-MB468 were analyzed by Immunoblot. β-actin was used as an internal control. **B**. Viability of MDA-MB157 cells after treatment with c-Met inhibitors (1, 10 and 100 μM) compared with 0.02% DMSO for 3 days was assessed by the amount of formazon formed by WST assay. Numerical values of test groups are shown with respect to 0.02% DMSO treated group. All data is represented as the mean ± S.D. of three independent experiments. **C**. c-Met phosphorylation level in MDA-MB157 was analyzed by immunoblot. MDA-MB157 cells were treated for 6h with Crizotinib (1.5 μM), Foretinib (1.5 μM), PHA-665752 (10 μM) and Tivantinib (0.5 μM).

**Figure 5:**
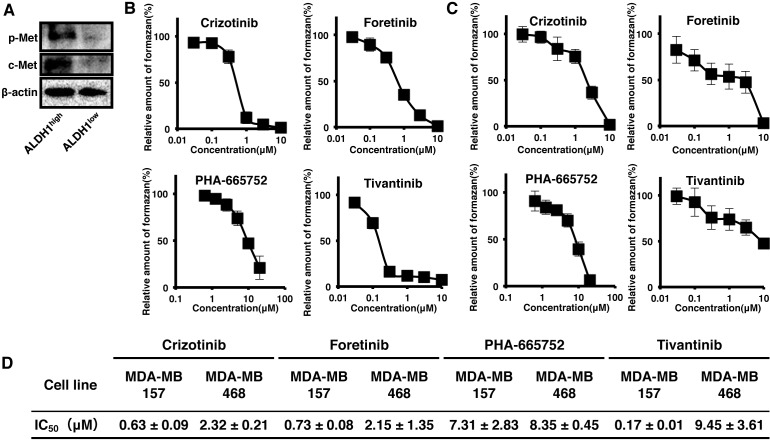
c-Met inhibitors suppressed viability of ALDH1^high^ cells derived from Basal-like type of breast cancer cells lines **A.** c-Met and Phosphorylated c-Met (p-Met) expression in ALDH1^high^ or ALDH1^low^ cells from MDA-MB157 were analyzed by Immunoblot. β-actin was used as an internal control. **B.-C.** Cell viability based on formation of formazon product as assessed by the WST-1 assay after 3 days of treatment with c-Met inhibitors, Crizotinib, Foretinib, PHA-665752, and Tivantinib (0.03, 0.1, 0.3, 1, 3 and 10 μM) in ALDH1^high^ cells derived from MDA-MB157 (B) and MDA-MB468 (C). Numerical values of test groups are shown with respect to 0.02% DMSO treated group. **D.** *In vitro* IC_50_ values of c-Met inhibitors in ALDH1^high^ cells derived from MDA-MB 157 and MDA-MB 468. All data is represented as the mean± S.D. from three independent experiments.

To reveal the role of c-Met in CSCs, we used MDA-MB157 and MDA-MB468 cell lines derived from human Basal-like type of breast cancer. c-Met protein was found to be highly expressed in MDA-MB157 cells in contrast to MDA-MB468 cells (Figure 4A). Next, we examined the effects of nine c-Met inhibitors on the viability of MDA-MB157 cells expressing higher c-Met protein (Table 2). Four c-Met inhibitors such as Crizotinib, Foretinib, PHA-665752 and Tivantinib strongly suppressed the viability of MDA-MB157 cells (Figure 4B). These results were consistent with the results of inhibition of c-Met phosphorylation level (indicating its activity) on treatment with c-Met inhibitors in MDA-MB157 cells (Figure 4C). Therefore, we next examined the inhibitory effects of these four c-Met inhibitors on the viability of ALDH1high cells derived from MDA-MB157 and MDA-MB468 cell lines. Isolated ALDH1^high^ cells derived from both MDA-MB157 and MDA-MB468 cell lines showed CSCs properties such as self-renewal, multi-differentiation, and tumorigenesis (Supplementary Figure 2A and 2B) as previously reported [9]. Interestingly, both c-Met and p-Met expression is higher in ALDH1^high^ cells than ALDH1^low^ cells (Figure 5A). The result suggests that ALDH1^high^ cells have high activity of c-Met. The c-Met inhibitors except for Tivantinib suppressed viability of ALDH1^high^ cells in both cell lines. The 50% cell growth inhibitory concentrations (IC_50_) of Crizotinib, Foretinib, and PHA-665752 were found to be lower in MDA-MB157 cells expressing higher c-Met protein than that in MDA-MB468 cells expressing lower c-Met protein (Figure 5B-D). Interestingly, Tivantinib specifically suppressed the viability of ALDH1^high^ MDA-MB157 cells. These results suggest that c-Met is necessary for the viability of ALDH1 positive breast CSCs.

**Table 2:** List of c-Met inhibitors

Compound name	Action mechanism	Targets	Reference
NVP-BVU972	ATP competitive Met inhibitor	c-Met	43
Tivantinib	ATP non-competitive Met inhibitor	c-Met	41,42
BMS777607	ATP competitive Met inhibitor	c-Met, RON, Axl, TYRO3 and MER	44
AMG-208	ATP competitive Met inhibitor	c-Met and RON	45
Cabozantinib	ATP competitive Met inhibitor	c-Met, VEGFR, RETKIT, FLT3 and TIE2	46
Foretinib	ATP competitive Met inhibitor	c-Met, VEGFR, AXL, PDGFR, KIT, FLT3 and TIE2	47,48
PF-04217903	ATP competitive Met inhibitor	c-Met	49,50
Crizotinib	ATP competitive Met inhibitor	c-Met and ALK	51,52
PHA-665752	ATP competitive Met inhibitor	c-Met, RON, FLK1 and c-Abl	53,54

### c-Met inhibitors suppressed tumor-sphere formation of ALDH1 positive CSCs

**Figure 6:**
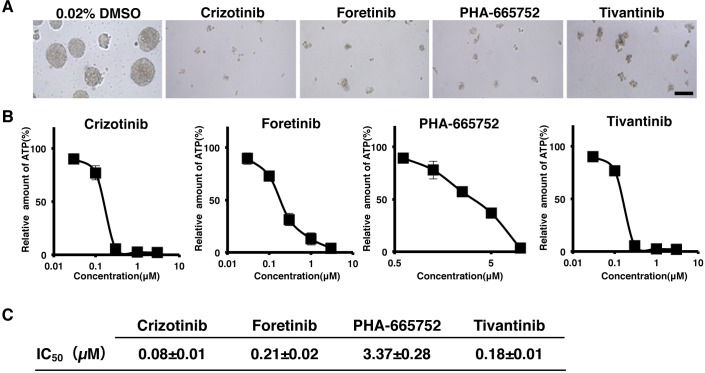
c-Met inhibitors suppressed tumor-sphere formation of ALDH1^high^ breast cancer cells **A.** Tumor-spheres of ALDH1^high^ cells derived from MDA-MB157 cells were incubated with c-Met inhibitors, Crizotinib (1 μM), Foretinib (1 μM), PHA-665752 (10 μM) and Tivantinib (1 μM) for 6 days. **B**. ATP level was assessed by the Cell-Titer Glo assay after treating tumor-spheres for 6 days with c-Met inhibitors, Crizotinib, Foretinib, Tivantinib (0.03, 0.1, 0.3, 1, and 3 μM) and PHA-665752 (0.6125, 1.25, 2.5, 5, and 10 μM). **C**. *In vitro* IC_50_ values with respect to decrease in ATP level on treatment with c-Met inhibitors. Numerical values of test groups are shown with respect to 0.02% DMSO treated group. All data is represented as the mean± S.D. from three independent experiments. Scale bar, 100 μm.

To investigate the role of c-Met in tumor formation of ALDH1 positive CSCs, we next examined the inhibitory effects of aforementioned inhibitory compounds on tumor-sphere formation in ALDH1 positive CSCs derived from MDA-MB157 *in vitro* system. As shown in Figure 6A and 6B, the inhibitory compounds were observed to suppress tumor-sphere formation. The IC_50_ values of these compounds for tumor-sphere formation were 0.18 μM (Crizotinib), 0.21 μM (Foretinib), 3.4μM (PHA-665752), and 0.18 μM (Tivantinib) (Figure 6C). These results suggest that c-Met is essential for tumor-sphere formation of ALDH1 positive CSCs in breast cancer cells.

Taken together with aforementioned results, it can be inferred that c-Met is specifically essential for cell viability and tumor-sphere formation of ALDH1 positive human breast CSCs.

## DISCUSSION

High expression of *c-Met* correlated with the expression of *ALDH1A3* in Basal-like type of breast cancer (Figure 1C). Since breast cancer stem cells exhibit a Basal-like phenotype [34], our result may thus provide new insights into the role of c-Met in ALDH1 positive CSCs of Basal-like type of breast cancer. It has been reported that knock-down of c-Met by siRNA and inhibitor treatment results in decrease of *ALDH1A3* gene expression and ALDEFLUOR activity in pancreatic cancer cell lines with high levels of c-Met [25]. Similarly, high c-Met expression and its activation are also suggested to be involved in the promotion of *ALDH1A3* gene expression in Basal-like type of breast cancer.

Several studies have reported that patients with higher expression of ALDH1 have poor prognosis in several cancers [9, 35]. In our study, Kaplan-Meier analysis revealed that patients with high *ALDH1A3* expression at tumor stage III-IV had poor outcome (*p* = 0.0049, Figure 3B). Similarly, patients expressing both *c-Met* and *ALDH1A3* at tumor stage III-IV had poor prognosis (*p* = 0.0065, Figure 3C). *c-Met* was found to be enriched in Basal-like type in comparison with other subtypes (Figure 1 and 2). However, patients expressing high *c-Met* and *ALDH1A3* in Basal-like type did not show poor outcome (*p* = 0.20; *n* = 199, Supplementary Figure 1D), which could be attributed to no correlation between *c-Met* and *ALDH1A3* in Basal-like type (Supplementary Figure 1A, *p* = 0.58, r = 0.039). In spite of no correlation between *c-Met* and *ALDH1A3* expression, major population of Basal-like type patients expressed high expression of *c-Met* and *ALDH1A3* (*n* = 93 in 199). Therefore, c-Met may play an important role in cancerous progression in Basal-like type. On the other hands, at tumor stage III-IV, of total analyzed patient samples (*n* = 124), each subtypes were distributed as follows; Luminal A (23%, *n* = 28), Luminal B (28%, *n* = 35), HER2-enriched (17%, n = 21), Claudin-low (16%, *n* = 20), Normal-like (5%, *n* = 6), and Basal-like (11%, *n* = 14). Therefore, co-expression of both *c-Met* and *ALDH1A3* at late tumor stages may contribute to poor clinical outcome not only in Basal-like but also in other subtypes. Since efficacy of chemotherapy at cancer spreading stage III-IV is extremely crucial, targeting c-Met in ALDH1 positive breast CSC may possibly decrease the severity of metastatic breast cancer and hence may lead to the survival of breast cancer patients. In addition, previous studies reported that ALDH1 is required for maintaining a drug-resistant cell subpopulation of stomach and breast cancer cells [36, 37, 38, 39]. Therefore, considering this, the drug resistance characteristics of breast cancers expressing *c-Met* and *ALDH1A3* should be analyzed in detail in the future for targeted cancer therapy.

We found that c-Met inhibitors suppressed cell viability and tumor-sphere formation of ALDH1^high^ cells (Figure 5 and 6). ALDH1 enzyme catalyzes the oxidation of aldehydes into corresponding acetic acids, and is involved in detoxification of toxic aldehyde intermediates produced in cancer cells. Recent studies reported that ALDH1 decreases ROS levels in various cancer cells and metabolizes toxic aldehydes formed by lipid peroxidation generated from intracellular lipids due to ROS [36, 40]. Since we observed strong correlation between ALDH1 and c-Met, use of c-Met inhibitors in ALDH1^high^ cells may have accumulated ROS and toxic aldehydes, which consequently may have lead to the induction of apoptosis in cancer cells.

Thus, it is suggested that c-Met plays an important role in ALDH1 positive breast CSCs. Although the ALDH1^high^ cells derived from MDA-MB157 and MDA-MB468 cells have been cultured *in vitro* in the presence of FBS, no loss in CSCs properties was observed (Supplementary Figure 2A and 2B). Since, loss of stem cell property due to long term culture of cells in *in vitro* in the presence of FBS has been reported, hence appropriate measures should be taken for long term culture of CSCs.

Among c-Met inhibitors, Crizotinib, Foretinib, PHA-665752 and Tivantinib, only Tivantinib specifically suppressed viability of high c-Met expressing MDA-MB157 cells as compared to low c-Met expressing MDA-MB468 cells (Figure 5). These results may depend on the inhibitory mechanisms of Tivantinib against c-Met activity. The c-Met inhibitors except Tivantinib are ATP competitor that docks to active site of c-Met kinase. ATP competitors generally inhibit the activity of other kinases and function of ATP associated molecules. In fact, Crizotinib, Foretinib and PHA-665752 strongly suppress the cell viability by inhibition of other kinases and ATP associated molecules (Table 2). On the other hand, Tivantinib, a non-ATP competitor, inhibits c-Met autophosphorylation and is highly selective for the inactive or non-phosphorylated form of c-Met by binding to ATP-binding cleft [41, 42]. Furthermore, the specific inhibitory effect of Tivantinib is profiled against 230 human kinases [41]. Non-ATP competitor such as Tivantinib binding to allosteric site must be explored further, as it may contribute to develop specific drugs targeting to c-Met in the future.

## CONCLUSION

In this study, we showed that high expression of *c-Met* correlated with the expression of *ALDH1A3* in Basal-like type of breast cancer. Patients with co-expression of *c-Met* and *ALDH1A3* at tumor stage III-IV showed poor clinical outcome. Furthermore, c-Met inhibitors suppressed the cell viability and tumor-sphere formation of ALDH1^high^ cells. These results suggest that c-Met is essential for the viability and tumor formation of ALDH1 positive CSCs. Therefore, c-Met protein is potential therapeutic target for ALDH1 positive breast CSCs.

## MATERIALS AND METHODS

### Cell culture

Human Basal-like type of breast cancer cell lines (MDA-MB157 and MDA-MB468) were obtained from American Type Culture Collection (ATCC, Manassas, VA, USA). Cell lines were grown in Dulbecco's Modified Eagle Medium (DMEM) medium supplemented with 10% fetal bovine serum (FBS) (Biosera, Dominican Republic) and penicillin/streptomycin. Cells were cultured at 37°C in a humidified atmosphere with 95% air/5% CO_2_.

### c-Met inhibitors and antibodies

c-Met inhibitors (AMG-208, BMS 777607, Cabozantinib, Crizotinib, Foretinib, NVP-BVU972, PF-04217903, PHA-665752, Tivantinib) were purchased from Namiki Inc. (Japan). All compounds dissolved in DMSO. Rabbit polyclonal c-Met antibody was purchased from Santa Cruz Inc. (USA). Rabbit monoclonal phospho-Met (Tyr1234/1235) antibody, HRP-conjugated anti-rabbit IgG and anti-mouse IgG were purchased from Cell Signaling Technology (USA). Mouse monoclonal β-actin antibody was obtained from Wako Inc. (Japan).

### Flow cytometry

Cells were exfoliated from culture dish by accutase (Innovative Cell Technology) and filtered through 40μm cell strainers (Greiner) to obtain single cells. The ALDH1^high^ cells were isolated from MDA-MB157 and MDA-MB468 cells by ALDEFLUOR assay kit (Stem Cell Technology) or AldeRed ALDH detection assay kit (MERCK) according to the manufacturer's instructions. Briefly, cells (2×10^6^) were incubated with the substrate for ALDH1 (5μL substrate/mL medium) for 30 min at 37°C. As a negative control for the ALDEFLUOR assay and AldeRed assay, cells were incubated with ALDH1 inhibitor, diethylaminobenzaldehyde (DEAB). The ALDH1^high^ cells were sorted by cell sorter (FACS AriaII, BD Bioscience) by taking the negative control into consideration. The analysis of CD10/EpCAM positive cells from MDA-MB157 and MDA-MB468 cells. Suspended MDA-MB468 cells (1×10^6^) were incubated with anti-CD10 (APC) (BD Bioscience) and anti-EpCAM (PE) (BD Bioscience) for 1hr on ice, after which the sample was washed with fresh FACS buffer (2%FBS in 1×PBS (-)). For this experiments, cells were analyzed using a FACS Calibur (BD Bioscience).

### WST-8 assay

Cells (3×10^5^/well) were seeded into 96 well culture plate (Sigma). One day post seeding, cells were treated with c-Met inhibitors for 3, 5, and 7 days. Cell viability was detected by WST-8 assay (Cell Counting Kit-8 (DOJINDO)). The formazan dye formed was measured by ARVO^TM^ MX (PerkinElmer) at 450 nm. Numerical values of test groups are shown with respect to 0.02% DMSO treated group.

### Immunoblotting

Cells were dissolved in RIPA buffer (50 mM Tris (pH 8.0), 150 mM NaCl, 0.5 w/v% sodium deoxycholate, 0.1 w/v% SDS, 1.0 w/v% Nonidet P-40 and protease inhibitor cocktail (Thermo Fisher)). Eight μg of whole cell lysate proteins was electrophoresed by SDS-PAGE (8% gel) and transferred to Immobilon-P Transfer Membrane (Millipore) or Immobilon-FL Transfer Membrane (Millipore). The transferred membranes were then blocked with 5% BSA in TTBS (25 mM Tris (pH 7.5), 140 mM NaCl, 2.5 mM KCl and 0.1% Tween 20) and incubated with the primary antibodies. The membranes were then probed with the horseradish peroxidase-conjugated secondary antibody. Specific signals were detected by chemiluminescence reagent, such as Immunostar LD/Immunostar Basic (Wako) using ChemiDoc MP (Bio-Rad).

### Tumor-sphere culture

Tumor-spheres were grown in DMEM culture medium containing 10% FBS, penicillin and streptomycin, 0.6% methyl cellulose (Wako), and 0.05 mM 2-mercaptoethanol (Sigma) at 37°C in a humidified atmosphere with 95% air/5% CO_2_. ALDH1^high^ cells (1x10^3^/well) were seeded and cultured in ultra low attachment 96-well plate (Greiner) for 6 days with or without inhibitory compounds. CellTiter-Glo^®^ luminescence assay (Promega) was performed by TR717 Micro plate Luminometer (TROPIX) using 96 well Micro-assay-plate (Greiner). Numerical values of test groups are shown with respect to 0.02% DMSO treated group.

### Analysis of gene expression data

Gene expression data was analyzed using METABRIC, Nature 2012 & Nat. Commun. 2016 dataset deposited in cBioPortal [28, 29, 30, 31]. Clinical data of the breast cancer patients used in our present study are summarized in Table S1. The median age at diagnosis was 61.1 years (aged 21.9 to 96.3 years). The dataset contains mRNA expression data of 1,904 primary breast tumor samples (patients) with details of breast cancer subtype (Normal-like, *n* = 140; Luminal A, *n* = 679; Luminal B, *n* = 461; HER2-enriched, *n* = 220; Claudin-low, *n* = 199; Basal-like, *n* = 199; Not classified, *n* = 6). We retrieved the mRNA expression (Z-scores) of genes and evaluated co-expression of *c-Met* and several stem cell markers in either all or each of the tumor stage groups. We defined the *c-Met* expression as follows; all stage patients were divided into c-Met^+^(c-Met mRNA expression Z-score>0, *n* = 837) and c-Met^-^(c-Met mRNA expression Z-score < 0, *n* = 1067) in Figure 1A, 1C and Table 1. Tumor stage III and IV patients were classified into c-Met^+^(c-Met mRNA expression Z-score>0, *n* = 74) and c-Met^-^(c-Met mRNA expression Z-score < 0, *n* = 50) in Figure 2A, 2B and Table 1. Pearson's correlation coefficiency was calculated for these expression levels for the subtypes in Figure 1B, 2C and Table 1. We also compared *c-Met* expression in all or stage III-IV groups. Quantitative variables were analyzed by Tukey's test. Data with *p* value less than 0.05 were considered significant. Survival curves were plotted by the Kaplan-Meier method and compared by the Gehan-Breslow generalized Wilcoxon test using BellCurve for Excel ver2.11. “High” and “low” were defined as the upper top 40% and the lower 60% of Z-score respectively, in several genes at stages III-IV breast cancer patients. Follow-up period after diagnosis ranged from 5.8 to 274.3 months stages III-IV breast cancer patients.

## SUPPLEMENTARY MATERIALS FIGURES AND TABLE



## References

[1] Ferlay J, Soerjomataram I, Dikshit R, Eser S, Mathers C, Rebelo M, Parkin DM, Forman D, Bray F (2015). Cancer incidence and mortality worldwide: sources, methods and major patterns in GLOBOCAN 2012. Int J Cancer.

[2] Parker JS, Mullins M, Cheang MC, Leung S, Voduc D, Vickery T, Davies S, Fauron C, He X, Hu Z, Quackenbush JF, Stijleman IJ, Palazzo J (2009). Supervised Risk Predictor of Breast Cancer Based on Intrinsic Subtypes. J Clin Oncol.

[3] Prat A, Pineda E, Adamo B, Galván P, Fernández A, Gaba L, Díez M, Viladot M, Arance A, Muñoz M (2015). Clinical implications of the intrinsic molecular subtypes of breast cancer. Breast.

[4] Sweeney C, Bernard PS, Factor RE, Kwan ML, Habel LA, Quesenberry CP, Shakespear K, Weltzien EK, Stijleman IJ, Davis CA, Ebbert MT, Castillo A, Kushi LH (2015). Intrinsic Subtypes from PAM50 Gene Expression Assay in a Population-Based Breast Cancer Cohort: Differences by Age, Race, and Tumor Characteristics. Cancer Epidemiol Biomarkers Prev.

[5] Lønning PE, Sørlie T, Børresen-Dale AL (2005). Genomics in breast cancer-therapeutic implications. Nature Reviews Clinical Oncology.

[6] Badve S, Dabbs DJ, Schnitt SJ, Baehner FL, Decker T, Eusebi V, Fox SB, Ichihara S, Jacquemier J, Lakhani SR, Palacios J, Rakha EA, Richardson AL (2011). Basal-like and triple-negative breast cancers: a critical review with an emphasis on the implications for pathologists and oncologists. Mod Pathol.

[7] Visvader JE, Lindeman GJ (2012). Cancer Stem Cells: Current Status and Evolving Complexities. Cell Stem Cell.

[8] Reya T, Morrison SJ, Clarke MF, Weissman IL (2001). Stem cells, cancer, and cancer stem cells. Nature.

[9] Ginestier C, Hur MH, Charafe-Jauffret E, Monville F, Dutcher J, Brown M, Jacquemier J, Viens P, Kleer CG, Liu S, Schott A, Hayes D, Birnbaum D (2007). ALDH1 is a marker of normal and malignant human mammary stem cells and a predictor of poor clinical outcome. Cell Stem Cell.

[10] Jiang F, Qiu Q, Khanna A, Todd NW, Deepak J, Xing L, Wang H, Liu Z, Su Y, Stass SA, Katz RL (2009). Aldehyde dehydrogenase 1 is a tumor stem cell-associated marker in lung cancer. Mol Cancer Res.

[11] Su Y, Qiu Q, Zhang X, Jiang Z, Leng Q, Liu Z, Stass SA, Jiang F (2010). ALDH1A1 Positive Cell Population Is Enriched in Tumorinitiating Cells and Associated with Progression of Bladder Cancer. Cancer Epidemiol Biomarkers Prev.

[12] Landen CN, Goodman B, Katre AA, Steg AD, Nick AM, Stone RL, Miller LD, Mejia PV, Jennings NB, Gershenson DM, Bast RC, Coleman RL, Lopez-Berestein G (2010). Targeting Aldehyde Dehydrogenase Cancer Stem Cells in Ovarian Cancer. Molecular Cancer Therapeutics.

[13] Cojoc M, Peitzsch C, Kurth I, Trautmann F, Kunz-Schughart LA, Telegeev GD, Stakhovsky EA, Walker JR, Simin K, Lyle S, Fuessel S, Erdmann K, Wirth MP (2015). Aldehyde Dehydrogenase Is Regulated by β-Catenin/TCF and Promotes Radioresistance in Prostate Cancer Progenitor Cells. Cancer Res.

[14] Marcato P, Dean CA, Liu RZ, Coyle KM, Bydoun M, Wallace M, Clements D, Turner C, Mathenge EG, Gujar SA, Giacomantonio CA, Mackey JR, Godbout R (2015). Aldehyde dehydrogenase 1A3 influences breast cancer progression via differential retinoic acid signaling. Mol Oncol.

[15] Marcato P, Dean CA, Pan D, Araslanova R, Gillis M, Joshi M, Helyer L, Pan L, Leidal A, Gujar S, Giacomantonio CA, Lee PW (2011). Aldehyde Dehydrogenase Activity of Breast Cancer Stem Cells is Primarily Due to Isoform ALDH1A3 and Its Expression is Predictive of Metastasis. Stem Cells.

[16] Nakshatri H, Srour EF, Badve S (2009). Breast Cancer Stem Cells and Intrinsic Subtypes: Controversies Rage On. Current Stem Cell Research & Therapy.

[17] Tsang JY, Huang YH, Luo MH, Ni YB, Chan SK, Lui PC, Yu AM, Tan PH, Tse GM (2012). Cancer stem cell markers are associated with adverse biomarker profiles and molecular subtypes of breast cancer. Breast Cancer Research and Treatment.

[18] Ricardo S, Vieira AF, Gerhard R, Leitão D, Pinto R, Cameselle-Teijeiro JF, Milanezi F, Schmitt F, Paredes J (2011). Breast cancer stem cell markers CD44, CD24 and ALDH1: expression distribution within intrinsic molecular subtype. J Clin Pathol.

[19] Organ SL, Tsao MS (2011). An overview of the c-MET signaling pathway. Therapeutic Advances in Medical Oncolog.

[20] Abounader R, Laterra J (2005). Scatter factor/hepatocyte growth factor in brain tumor growth and angiogenesis. Neuro-Oncology.

[21] Miller CT, Lin L, Casper AM, Lim J, Thomas DG, Orringer MB, Chang AC, Chambers AF, Giordano TJ, Glover TW, Beer DG (2006). Genomic amplification of MET with boundaries within fragile site FRA7G and upregulation of MET pathways in esophageal adenocarcinoma. Oncogene.

[22] Okuda K, Sasaki H, Yukiue H, Yano M, Fujii Y (2008). Met gene copy number predicts the prognosis for completely resected non-small cell lung cancer. Cancer Sci.

[23] Liu X, Newton RC, Scherle PA (2010). Developing c-MET pathway inhibitors for cancer therapy: progress and challenges. Trends Mol Med.

[24] Rath P, Lal B, Ajala O, Li Y, Xia S, Kim J, Laterra J (2013). In Vivo c-Met Pathway Inhibition Depletes Human Glioma Xenografts of Tumor-Propagating Stem-Like Cells. Transl Oncol.

[25] Kim IG, Lee JH, Kim SY, Kim JY, Cho EW (2014). Fibulin-3 negatively regulates ALDH1 via c-MET suppression and increases γ-radiation-induced sensitivity in some pancreatic cancer cell lines. Biochem Biophys Res Commun.

[26] Lim YC, Kang HJ, Moon JH (2014). c-Met pathway promotes self-renewal and tumorigenecity of head and neck squamous cell carcinoma stem-like cell. Oral Oncology.

[27] Noguchi K, Eguchi H, Konno M, Kawamoto K, Nishida N, Koseki J, Wada H, Marubashi S, Nagano H, Doki Y, Mori M, Ishii H (2015). Susceptibility of pancreatic cancer stem cells to reprogramming. Cancer Sci.

[28] Curtis C, Shah SP, Chin SF, Turashvili G, Rueda OM, Dunning MJ, Speed D, Lynch AG, Samarajiwa S, Yuan Y, Gräf S, Ha G, Haffari G (2012). The genomic and transcriptomic architecture of 2,000 breast tumours reveals novel subgroups. Nature.

[29] Pereira B, Chin SF, Rueda OM, Vollan HK, Provenzano E, Bardwell HA, Pugh M, Jones L, Russell R, Sammut SJ, Tsui DW, Liu B, Dawson SJ (2016). The somatic mutation profiles of 2,433 breast cancers refine their genomic and transcriptomic landscapes. Nat Commun.

[30] Cerami E, Gao J, Dogrusoz U, Gross BE, Sumer SO, Aksoy BA, Jacobsen A, Byrne CJ, Heuer ML, Larsson E, Antipin Y, Reva B, Goldberg AP (2012). The cBio Cancer Genomics Portal: an open platform for exploring multidimensional cancer genomics data. Cancer Dicov.

[31] Gao J, Aksoy BA, Dogrusoz U (2013). Integrative analysis of complex cancer genomics and clinical profiles using the cBioPortal. Sci Signal.

[32] Nautiyal J, Kanwar SS, Yu Y, Majumdar AP (2011). Combination of dasatinib and curcumin eliminates chemo-resistant colon cancer cells. J Mol Signal.

[33] Sun B, Liu R, Xiao ZD, Zhu X (2012). c-MET protects breast cancer cells from apoptosis induced by sodium butyrate. PLoS One.

[34] Farrar William L (2010). Cancer Stem Cell.

[35] Charafe-Jauffret E, Ginestier C, Iovino F, Tarpin C, Diebel M, Esterni B, Houvenaeghel G, Extra JM, Bertucci F, Jacquemier J, Xerri L, Dontu G, Stassi G (2010). ALDH1-positive cancer stem cells mediate metastasis and poor clinical outcome in inflammatory breast cancer. Clin Cancer Res.

[36] Raha D, Wilson TR, Peng J, Peterson D, Yue P, Evangelista M, Wilson C, Merchant M, Settleman J (2014). The Cancer Stem Cell Marker Aldehyde Dehydrogenase Is Required to Maintain a Drug-Tolerant Tumor Cell. Cancer Res.

[37] Nishikawa S, Konno M, Hamabe A, Hasegawa S, Kano Y, Ohta K, Fukusumi T, Sakai D, Kudo T, Haraguchi N, Satoh T, Takiguchi S, Mori M (2013). Aldehyde dehydrogenase high gastric cancer stem cells are resistant to chemotherapy. Int J Oncol.

[38] Croker AK, Allan AL (2012). Inhibition of aldehyde dehydrogenase (ALDH) activity reduces chemotherapy and radiation resistance of stem-like ALDHhiCD44+ human breast cancer cells. Breast Cancer Res Treat.

[39] Tanei T, Morimoto K, Shimazu K, Kim SJ, Tanji Y, Taguchi T, Tamaki Y, Noguchi S (2009). Association of Breast Cancer Stem Cells Identified by Aldehyde Dehydrogenase 1 Expression with Resistance to Sequential Paclitaxel and Epirubicin-Based Chemotherapy for Breast Cancers. Clin Cancer Res.

[40] Singh S, Brocker C, Koppaka V, Chen Y, Jackson BC, Matsumoto A, Thompson DC, Vasiliou V (2013). Aldehyde Dehydrogenases in Cellular Responses to Oxidative/electrophilic Stress. Free Radic Biol Med.

[41] Munshi N, Jeay S, Li Y, Chen CR, France DS, Ashwell MA, Hill J, Moussa MM, Leggett DS, Li CJ (2010). ARQ 197, a Novel and Selective Inhibitor of the Human c-Met Receptor Tyrosine Kinase with Antitumor Activity. Mol Cancer Ther.

[42] Eathiraj S, Palma R, Volckova E, Hirschi M, France DS, Ashwell MA, Chan TC (2011). Discovery of a novel mode of protein kinase inhibition characterized by the mechanism of inhibition of human mesenchymal-epithelial transition factor (c-Met) protein autophosphorylation by ARQ197. J. Biol. Chem.

[43] Tiedt R, Degenkolbe E, Furet P, Appleton BA, Wagner S, Schoepfer J, Buck E, Ruddy DA, Monahan JE, Jones MD, Blank J, Haasen D, Drueckes P (2011). A drug resistance screen using a selective MET inhibitor reveals a spectrum of mutations that partially overlap with activating mutations found in cancer patients. Cancer Res.

[44] Schroeder GM, An Y, Cai ZW, Chen XT, Clark C, Cornelius LA, Dai J, Gullo-Brown J, Gupta A, Henley B, Hunt JT, Jeyaseelan R, Kamath A (2009). Discovery of N-(4-(2-amino-3-chloropyridin-4-yloxy)-3-fluorophenyl)-4-ethoxy-1-(4-fluorophenyl)-2-oxo-1,2-dihydropyridine-3-carboxamide (BMS-777607), a selective and orally efficacious inhibitor of the Met kinase superfamily. J Med Chem.

[45] Albrecht Brian K, Harmange Jean-Christophe, Bauer David, Berry Loren, Bode Christiane, Boezio Alessandro A, Chen April, Choquette Deborah, Dussault Isabelle, Fridrich Cary, Hirai Satoko, Hoffman Doug, Larrow Jay F (2008). Discovery and Optimization of Triazolopyridazines as Potent and Selective Inhibitors of the c-Met Kinase. J. Med. Chem.

[46] Katayama R, Kobayashi Y, Friboulet L, Lockerman EL, Koike S, Shaw AT, Engelman JA, Fujita N (2015). Cabozantinib overcomes crizotinib resistance in ROS1 fusion positive cancer. Clin Cancer Res.

[47] Liu L, Greger J, Shi H, Liu Y, Greshock J, Annan R, Halsey W, Sathe GM, Martin AM, Gilmer TM (2009). Novel mechanism of Lapatinib resistance in HER2-positive breast tumor cells: activation of AXL. Cancer Res.

[48] Qian F, Engst S, Yamaguchi K, Yu P, Won KA, Mock L, Lou T, Tan J, Li C, Tam D, Lougheed J, Yakes FM, Bentzien F (2009). Inhibition of tumor cell growth, invasion, and metastasis by EXEL-2880 (XL880, GSK1363089), a novel inhibitor of HGF and VEGF receptor tyrosine kinases. Cancer Res.

[49] Cui JJ, McTigue M, Nambu M, Tran-Dubé M, Pairish M, Shen H, Jia L, Cheng H, Hoffman J, Le P, Jalaie M, Goetz GH, Ryan K (2012). Discovery of a novel class of exquisitely selective mesenchymal-epithelial transition factor (c-MET) protein kinase inhibitors and identification of the clinical candidate 2-(4-(1-(quinolin-6-ylmethyl)-1H-[1,2,3]triazolo[4,5-b]pyrazin-6-yl)-1H-pyrazol-1-yl)ethanol (PF-04217903) for the treatment of cancer. J Med Chem.

[50] Zou HY, Li Q, Lee JH, Arango ME, Burgess K, Qiu M, Engstrom LD, Yamazaki S, Parker M, Timofeevski S, Cui JJ, McTigue M, Los G (2012). Sensitivity of Selected Human Tumor Models to PF-04217903, a Novel Selective c-Met Kinase Inhibitor. Mol Cancer Ther.

[51] Zou HY, Li Q, Lee JH, Arango ME, McDonnell SR, Yamazaki S, Koudriakova TB, Alton G, Cui JJ, Kung PP, Nambu MD, Los G, Bender SL (2007). An orally available small-molecule inhibitor of c-Met, PF-2341066, exhibits cytoreductive antitumor efficacy through antiproliferative and antiangiogenic mechanisms. Cancer Res.

[52] Christensen JG, Zou HY, Arango ME, Li Q, Lee JH, McDonnell SR, Yamazaki S, Alton GR, Mroczkowski B, Los G (2007). Cytoreductive antitumor activity of PF-2341066, a novel inhibitor of anaplastic lymphoma kinase and c-Met, in experimental models of anaplastic large-cell lymphoma. Mol Cancer Ther.

[53] Christensen JG, Schreck R, Burrows J, Kuruganti P, Chan E, Le P, Chen J, Wang X, Ruslim L, Blake R, Lipson KE, Ramphal J, Do S (2003). A Selective Small Molecule Inhibitor of c-Met Kinase Inhibits c-Met-Dependent Phenotypes in Vitro and Exhibits Cytoreductive Antitumor Activity in Vivo. Cancer Res.

[54] Smolen GA, Sordella R, Muir B, Mohapatra G, Barmettler A, Archibald H, Kim WJ, Okimoto RA, Bell DW, Sgroi DC, Christensen JG, Settleman J, Haber DA (2006). Amplification of MET may identify a subset of cancers with extreme sensitivity to the selective tyrosine kinase inhibitor PHA-665752. PNAS.

