# Supplementary material for: Correlation between c-Met and ALDH1 contributes to the survival and tumor-sphere formation of ALDH1 positive breast cancer stem cells and predicts poor clinical outcome in breast cancer
Source: Genes Cancer. 2017 Jul;8(7-8):628–39. doi: 10.18632/genesandcancer.148 (PMC5620008; doi:10.18632/genesandcancer.148)
Supplement: Supplementary file 1 [file ganc-08-628-s001.pdf]

Correlation between c-Met and ALDH1 contributes to the survival and tumor-sphere formation of ALDH1 positive breast cancer stem cells and predicts poor clinical outcome in breast cancer – Nozaki et al.

Supplementary Materials

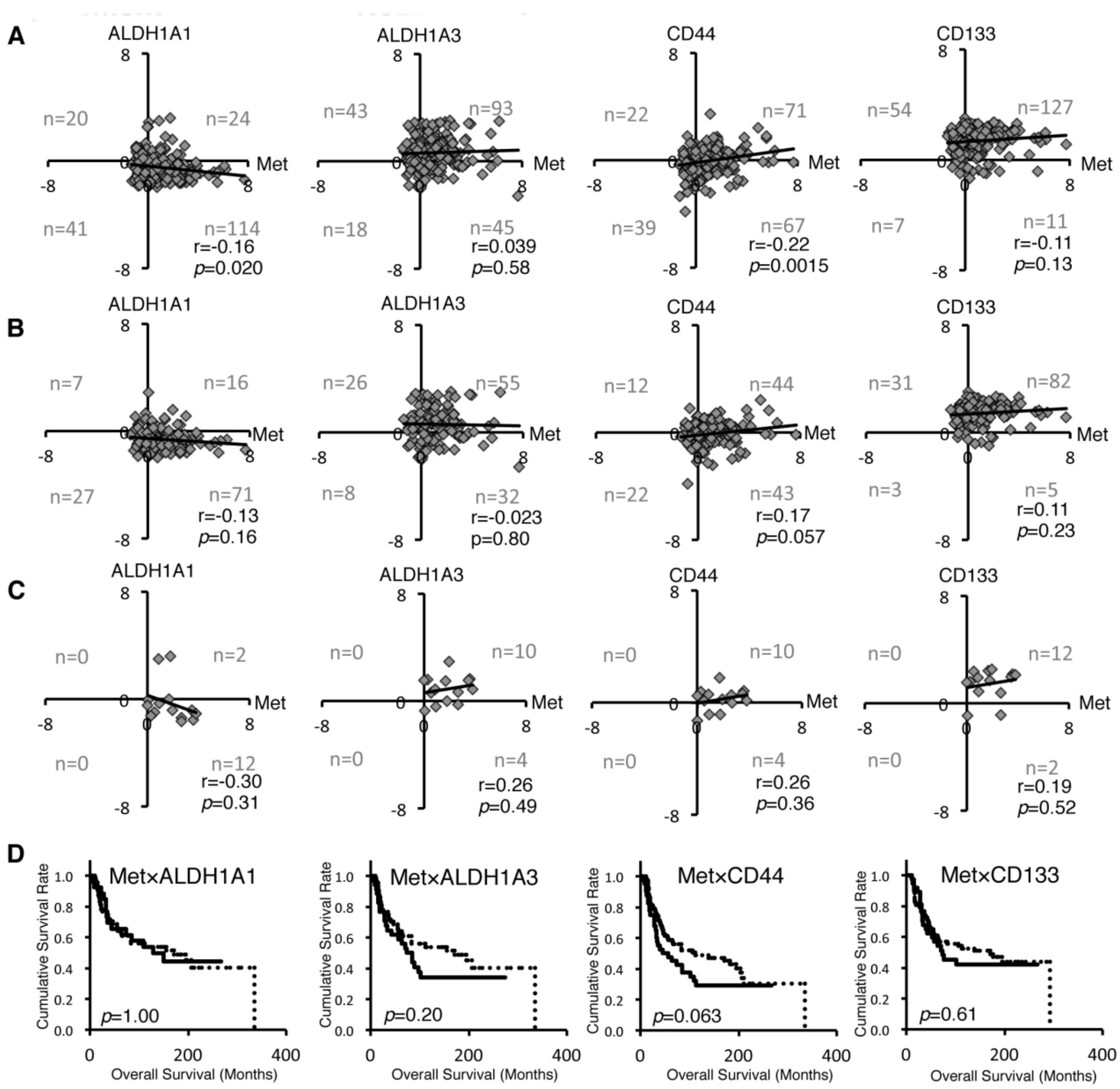

Supplementary Figure 1: No correlation between *c-Met* and *ALDH1A1, ALDH1A3, CD44, or CD133* in Basal-like type. (A-C) Scatter plots of *c-Met* with *ALDH1A1, ALDH1A3, CD44,* and *CD133* in Basal-like type of breast cancer patients at all tumor stage (A), stage 0-□ (B) and stage □-□(C). The coefficient of correlation (r) and the *p* value (*p*) are indicated. (D) Kaplan-Meier Survival curves of Basal-like type at tumor stage III-IV in terms of *c-Met* and *ALDH1A1*, *ALDH1A3*, *CD44* or *CD133* expression.

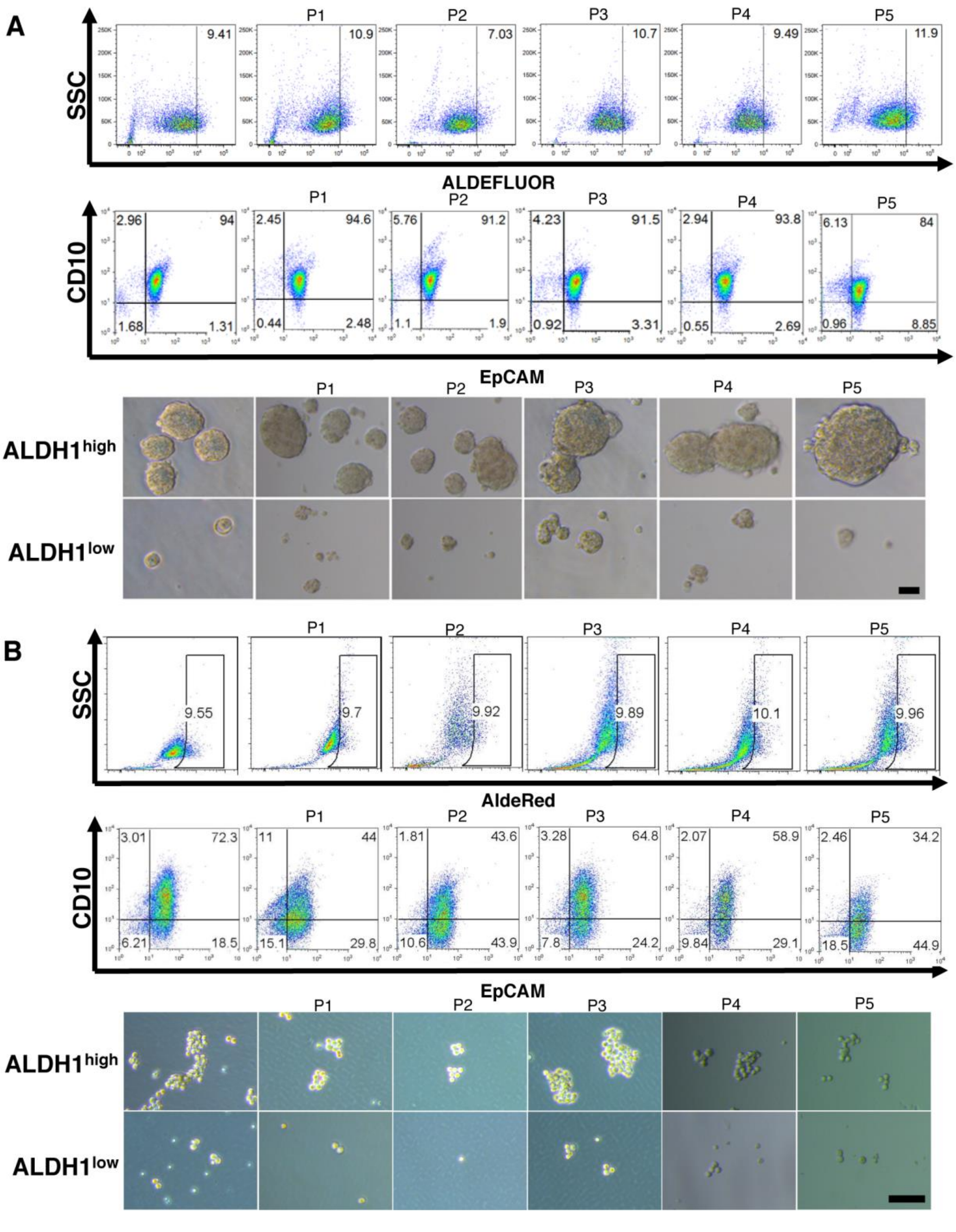

Supplementary Figure 2: Serial passage of ALDH1^{high} cells isolated from MDA-MB 157 (A) and MDA-MB 468 (B) cell lines retain CSCs properties. Upper panels in (A) and (B) were shown the

results of ALDEFLUOR assay (A) or AldeRed assay in serial passages. Middle panels in (A) and (B) were shown the expression of CD10 (marker as myoepithelial cells) and EpCAM (marker as luminal cells) in serial passages. Bottom panels in (A) and (B) were shown the results of tumor-sphere culture by using ALDH1^{high} cells and ALDH1^{low} cells in serial passage. Cells were cultured for 5-9 days (A) and 7-21 days (B). Scale bar, 50μm.

Table S1: Clinicopathological data

Variable	n=1904	(%)
**Age 61.1±13.0 (21.9-96.3)**		
≧61.1	988	(51.9)
＜61.1	916	(48.1)
**Gender**		
Male	0	0.0
Female	1904	#####
**Tumor size**		
≧5cm	142	(7.5)
＜5cm	1744	(91.6)
Not informative	18	(0.9)
**Tumor Stage**		
Stage 0	4	(0.2)
Stage Ⅰ	475	(24.9)
Stage Ⅱ	800	(42.0)
Stage Ⅲ	115	(6.0)
Stage Ⅳ	9	(0.5)
Not informative	501	(26.3)
**Histological Subtype**		
DCIS	2	(0.1)
IDC	1727	(90.7)
ILC	141	(7.4)

BENIGN	1	(0.1)
Invasive Tumor	9	(0.5)
Mixed Nst and a special type	3	(0.2)
Other	10	(0.5)
Other Invasive	9	(0.5)
Not informative	2	(0.1)
Pam50 + Claudin-low subtype		
Normal-like	140	(7.4)
Luminal A	679	(35.7)
Luminal B	461	(24.2)
HER2-enriched	220	(11.6)
Claudin-low	199	(10.5)
Basal-like	199	(10.5)
Not informative	6	(0.3)
